# Thermal Insulation of YSZ and Erbia-Doped Yttria-Stabilised Zirconia EB-PVD Thermal Barrier Coating Systems after CMAS Attack

**DOI:** 10.3390/ma13194382

**Published:** 2020-10-01

**Authors:** Germain Boissonnet, Christine Chalk, John R. Nicholls, Gilles Bonnet, Fernando Pedraza

**Affiliations:** 1 Laboratoire des Sciences de l’Ingénieur pour l’Environnement (LaSIE UMR-7356 CNRS), Université de La Rochelle, Avenue Michel Crépeau, 17042 La Rochelle, France; gbonnet@univ-lr.fr (G.B.); fpedraza@univ-lr.fr (F.P.); 2 Surface Engineering and Nanotechnology Institute (SENTi), Cranfield University, College Rd., Wharley End, Bedford MK43 0AL, UK; c.chalk@cranfield.ac.uk (C.C.); J.R.Nicholls@cranfield.ac.uk (J.R.N.)

**Keywords:** thermal barrier coatings (TBCs), electron-beam physical vapour deposition (EB-PVD), yttria-stabilised zirconia (YSZ), Erbia-yttria co-stabilised zirconia, thermal diffusivity

## Abstract

The impact of small deposits of calcium–magnesium–aluminium silicates (CMAS) on the top of thermal barrier coatings (TBCs) made of yttria-stabilised zirconia (YSZ) produced via electron-beam physical vapour deposition (EB-PVD) is shown to play a role in the microstructural and chemical stability of the coatings; hence, it also affects the thermal insulation potential of TBCs. Therefore, the present work investigates the degradation potential of minor CMAS deposits (from 0.25 to 5 mg·cm^−2^) annealed at 1250 °C for 1 h on a novel Er_2_O_3_-Y_2_O_3_ co-stabilised ZrO_2_ (ErYSZ) EB-PVD TBC, which is compared to the standard YSZ coating. Due to the higher reactivity of ErYSZ coatings with CMAS, its penetration is limited in comparison with the standard YSZ coatings, hence resulting in a better thermal insulation of the former after ageing.

## 1. Introduction

Thermal barrier coatings (TBCs) allow both better performance and an extension of component durability via the protection of superalloy components in the hottest sections of gas turbines [1,2]. These multilayer coatings comprise a metallic bond coating (BC) that provides resistance to oxidation while growing a thin oxide known as the thermally grown oxide (TGO), which is also responsible for the adherence of the ceramic top coating (TC). The latter acts as a thermal insulator between the substrate and the hot gases via a highly porous structured layer of zirconia (ZrO_2_) stabilised with 6-9 wt.% yttria (Y_2_O_3_) [3]. The electron-beam physical vapour deposition (EB-PVD) process for the ceramic top coating manufacturing leads to a feather-like segmented columnar structure that provides high strain compliance and hence a superior thermal shock resistance for application to aeroengine blades [4]. However, to reach higher efficiencies and performance of turbine engines, the inlet gas temperature must increase further. This ever-increasing demand leads to the constant need to search for new solutions with, for instance, an interest in developing top coatings with even greater thermal insulation potential [5]. For this purpose, coating materials are moving toward complex compositions such as pyrochlore structures based on zirconates (e.g., La_2_Zr_2_O_7_, Gd_2_Zr_2_O_7_) as well as various zirconia alloys via multicomponent stabilisation. To some extent, doping YSZ with several other additives such as NiO, Nd_2_O_3_, Er_2_O_3_, Yb_2_O_3_, and Gd_2_O_3_ allowed an enhancement of TBCs performances via a reduction of the thermal conductivity of the YSZ material that would thus permit reaching higher temperatures [6].

However, the increase of the temperature in the hot-gas path of turbine engines brings in the corrosion of components by calcium–magnesium–aluminium–silicates (CMAS) [7]. The CMAS-induced phenomenon occurs because the siliceous particles ingested in the engines melt and penetrate the porous ceramic coatings. This leads to a progressive degradation of their insulating capacity via sintering and spallation mechanisms that further contribute to a reduction in the lifetime of the coated components [3,8,9,10,11]. However, the introduction of rare-earth elements in the composition of TBCs was shown to be an effective mitigation solution against this. Based on the chemical reactivity of the TBC material in contact with the CMAS melt, recent strategies focus on preventing the penetration of the molten silicates via the precipitation of stable phases as soon as the dissolution of the TBC material starts. For instance, Aygun et al. showed that co-doping of YSZ with Al_2_O_3_ and TiO_2_ promoted the crystallisation of the anorthite phase CaAl_2_Si_2_O_8_ via the local enrichment of Al_2_O_3_ into the CMAS melt [12]. More recently, rare-earth (RE) zirconates used as TBC material (RE = Gd [13], Y [14,15], Yb [15], Sm [16,17], Nd [18], La [19]) were shown to foster the rapid crystallisation of stable apatite and fluorite phases as soon as the CMAS melt starts to dissolve the ceramic coating [7,20].

Among the different potential materials for the future of TBCs, Er-doped coatings showed promising results in terms of thermal insulation potential [21]. However, their potential evolution when exposed to CMAS and the resulting impact on the thermal insulating properties are yet to be investigated. Therefore, in this work, the thermal insulation potential and the resistance to CMAS attack of erbia-yttria co-stabilised zirconia EB-PVD coatings have been compared to those of the state-of-the-art yttria-stabilised zirconia. Both coatings were synthesised on Ni-based alloys and exposed to high temperature to evaluate the impact of the microstructural transformations on the thermal insulation properties.

## 2. Materials and Methods

Due to availability, Inconel 600 substrate (15.5 Cr, 8.0 Fe, 0.5 Mn, <0.5 Cu, <0.5 Si, wt.%, bal. Ni) was selected for the elaboration of the different samples of the study. After aluminising via a Chemical Vapour Deposition (CVD) process, 10 × 10 mm^2^ coupons were coated either with the standard yttria-stabilised zirconia (8YSZ) or with the Er_2_O_3_-Y_2_O_3_ co-stabilised ZrO_2_ (ErYSZ) ceramic coatings using a composition of 8YSZ (ZrO_2_—8 wt.% Y_2_O_3_ (4 mol.% Y_2_O_3_)) and ErYSZ (ZrO_2_—4 mol.% Y_2_O_3_—4 mol.% Er_2_O_3_), respectively. The coatings were deposited at a rate of 1.6–2.1 µm·min^−1^ for 75 min to achieve coatings of ≈150 µm.

For the corrosion tests, a CMAS mixture whose composition is representative of low melting deposits observed in CMAS-attacked aero-engines (36 CaO, 6 MgO, 5 Al_2_O_3_, 52 SiO_2_, and 1 Fe_2_O_3_ in mol.%) was obtained by melting a mixture of the different individual oxides at 1400 °C for 4 h in a Pt-10Rh crucible followed by crushing the resulting glass in a mortar after cooling [7,12,22]. The melting operation was operated a second time to improve the glass homogeneity. Following the same technique described in previous studies [22], CMAS deposits of 0.25, 1, and 5 mg·cm^−2^ were applied on both types of coatings (Table 1) and heated at 1250 °C for 1 h. Thereafter, the samples were slowly cooled down in the furnace until room temperature to avoid spallation.

Microstructural changes were investigated using scanning electron microscopy (SEM: FEI Quanta 200F, La Rochelle, France) coupled to energy dispersive spectrometry (EDS: EDAX detector, La Rochelle, France) for local analysis of the composition. Raman micro-spectrometry (Jobin Yvon LabRam HR800, λ_laser_ ≈ 632 nm, La Rochelle, France) was employed to follow the structural changes of the ceramic coatings on the cross-section of the coatings, while X-ray diffraction (XRD, Bruker AXS D8 Advance, La Rochelle, France) was conducted to determine the crystal structure of the surface compounds using the λ_Cu_ radiation in *θ*–2*θ* mode. The thermal insulation potential of the TBCs was assessed using thermal diffusivity measurements from room temperature until 1100 °C with a laser-flash apparatus (Linseis LFA1600, InSb infrared detector, La Rochelle, France). The measurements were made under vacuum (1 × 10^−2^ mbar) every 100 °C with 5 shots per sample at each temperature step. Then, the contribution of the coating was retrieved from the measurements of the multilayer samples (coatings + substrate) using calculations based on a 2-layer model. The thickness of each sample was precisely measured with a digital calliper (10^−3^ mm), while the surfaces were coated with a thin sputtered layer of gold and graphite prior to the laser-flash measurements to foster absorption of the laser and heat emissivity. For the multilayer calculations, the thickness of each individual layer was assessed from the cross-sectional micrographs. To compare the data to other studies, the thermal conductivity was assessed based on Equation (1):

(1) $$k\left(T\right)=\alpha \left(T\right)\times \rho \left(T\right)\times C_{p}\left(T\right)$$

where k is the thermal conductivity, α the thermal diffusivity, ρ is the density and $C_{p}$ is the specific heat capacity of the material at a given temperature. The specific heat capacity of 8YSZ and of ErYSZ compositions was calculated from the heat capacity values of the constituent oxides according to the Neumann–Kopp rule [23,24]. The density of the coatings was estimated from porosity measurements using SEM image analysis and the theoretical value of bulk density. Therefore, the calculated thermal conductivity results from an estimation of the different thermophysical properties. The errors are already significant when calculating the thermal diffusivity of a thin coating (≈150 µm) deposited on a thick substrate (1.5 mm) using a 2-layer model that also neglects interfacial contributions, leading to potential inaccuracies. In addition, the appearance of new phases after the CMAS attack may also modify the specific thermophysical properties, as Moskal et al. demonstrated by sulphate attack of their plasma sprayed TBCs [25]. Therefore, the calculated thermal conductivity values should be considered in a comparative way. 

## 3. Results

### 3.1. EB-PVD Coatings without CMAS

**Figure 1:**
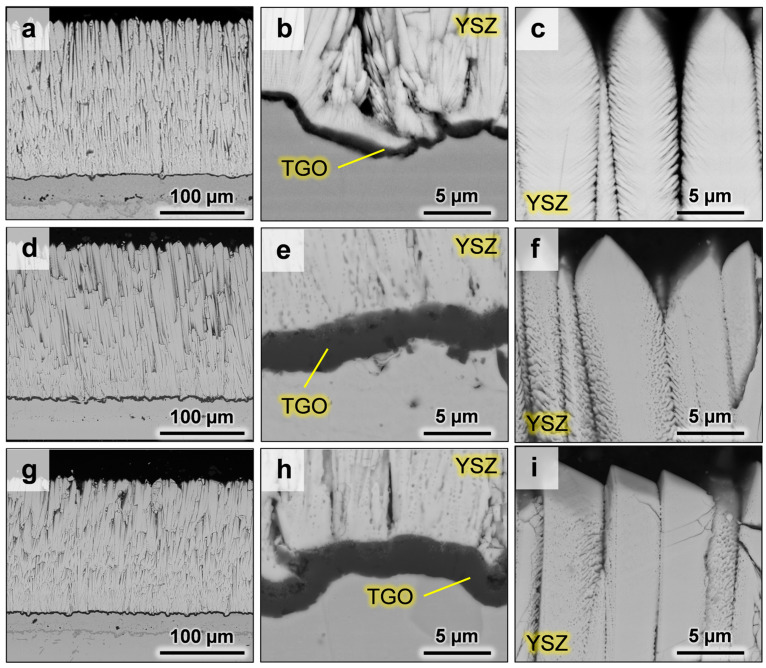
SEM micrographs of the electron-beam physical vapour deposition (EB-PVD) standard yttria-stabilised zirconia (8YSZ) coatings in the as-deposited conditions with (**a**–**c**) (similar for both standard and Er_2_O_3_-doped coatings) and after ageing in air at 1250 °C for 1 h with (**d**–**f**) for the standard 8YSZ coating and (**g**–**i**) for the ErYSZ coating.

Figure 1 presents the microstructural features of the as-deposited coatings (Figure 1a–c) compared with standard 8YSZ (Figure 1d–f) and ErYSZ coatings (Figure 1g–i) annealed at 1250 °C for 1 h.

Both the standard 8YSZ and the ErYSZ coatings present the typical columnar morphology of EB-PVD ceramic top coatings attached to a thin (≈0.6 µm) *α*-Al_2_O_3_ grown oxide layer on a *β*-NiAl bond coating of about 30 µm in the as-deposited conditions (Figure 1a). With annealing at 1250 °C for 1 h, the *α*-Al_2_O_3_ layer grows until ≈2 µm in both coatings (Figure 1e,h), and the fine lamellar porosity of the feather-like TBC morphology tends to sinter to form smaller globular pores (Figure 1f,i). For the 8YSZ coating (Figure 1f), the intra-lamellar gaps started to close. In the case of the ErYSZ ceramic (Figure 1i), the stiffening that may have occurred during ageing resulted in the appearance of cracks in the columns.

**Figure 2:**
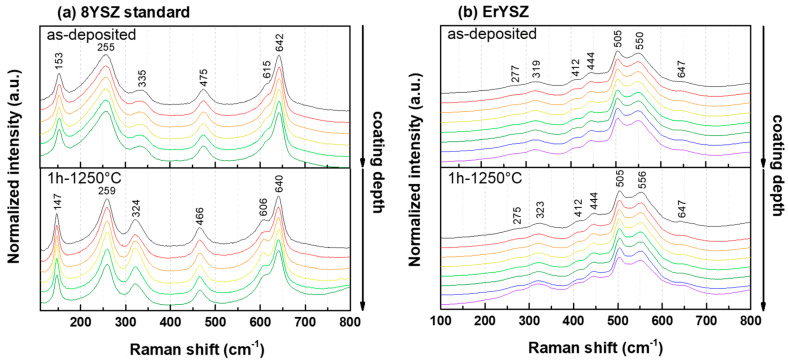
Raman spectra of (**a**) the 8YSZ standard and (**b**) Er_2_O_3_-Y_2_O_3_ co-stabilised ZrO_2_ (ErYSZ) coatings in their as-deposited conditions and after annealing in air at 1250 °C for 1 h.

Figure 2 shows the phase composition along the cross-section of both coatings in the as-deposited conditions and after annealing in air at 1250 °C for 1 h measured by Raman micro-spectrometry.

For the standard 8YSZ coating, the Raman spectra show that the stabilised tetragonal phase of zirconia (*t*′-ZrO_2_) is the only one detected for both as-deposited and annealed coatings, with the characteristic bands of tetragonal ZrO_2_ at ≈153, 255, 335, 475, 615, and 642 cm^−1^ [26,27]. After annealing, the Raman bands tend to sharpen and are slightly shifted compared to the as-deposited coating. For the ErYSZ coatings, the same bands are observed for both as-deposited and annealed conditions. The bands observed at ≈275, 320, 410, 445, 505, 550, and 650 cm^−1^ have also been observed for ErYSZ powders in previous studies [21,28]. He et al. explained that the Raman bands of ZrO_2_ overlap with the fluorescence bands of Er^3+^, thus preventing a clear identification of the phases of zirconia [28].

### 3.2. EB-PVD Coatings Corroded by CMAS

**Figure 3:**
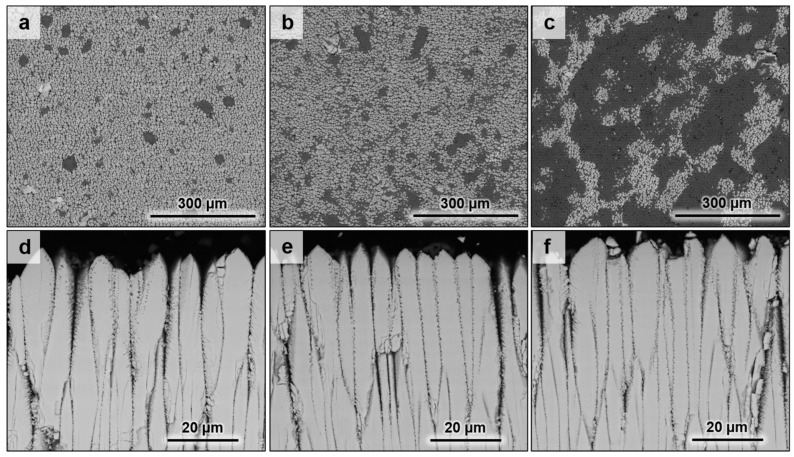
SEM micrographs of the standard 8YSZ coating with calcium–magnesium–aluminium silicates (CMAS) deposits of (**a**,**d**) 0.25 mg·cm^−2^, (**b**,**e**) 1 mg·cm^−2^, and (**c**,**f**) 5 mg·cm^−2^ annealed in air at 1250 °C for 1 h.

On the surface of the 8YSZ standard coatings annealed at 1250 °C with CMAS in Figure 3a–c, a dark phase associated with melted CMAS covers an increasing surface area of the coating with increasing the amount of deposited CMAS.

**Figure 4:**
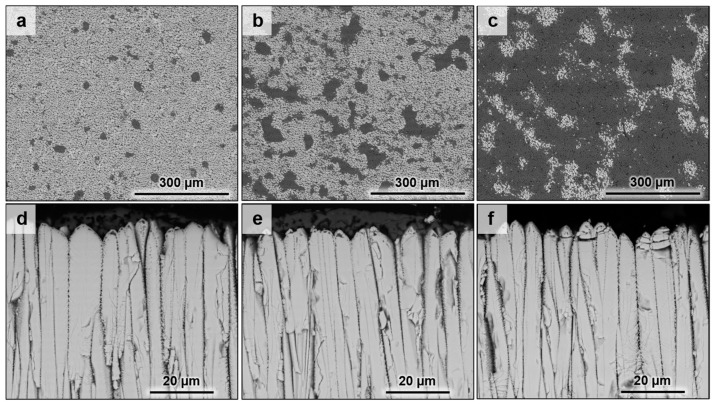
SEM micrographs of the co-stabilised ErYSZ coating with CMAS deposits of (**a**,**d**) 0.25 mg·cm^−2^, (**b**,**e**) 1 mg·cm^−2^, and (**c**,**f**) 5 mg·cm^−2^ annealed in air at 1250 °C for 1 h.

This residual CMAS means that the melt did not fully penetrate the ceramic coatings even at these low CMAS dose rates. On the associated cross-sections (Figure 3d–f), the microstructure of the upper part of the columns looks corroded along with an apparent sintering between the columns. This corrosion is linked with the appearance of fine bubble-shaped pores on the edges of the columns when the coating material interacted with the CMAS melt. Then, by following the microstructural transformations along the columns, it is observed that the depth of penetration of the melt increases with the amount of CMAS. Thus, this penetration led to transformations of the edges of the columns as well as sintering due to the sealing of the inter-columnar gap by the CMAS. For the ErYSZ coatings, a similar distribution of the residual CMAS is observed on the surface of the coatings (Figure 4a–c).

However, the columns appear less corroded on the cross-section micrographs of ErYSZ coatings than on those of standard 8YSZ (Figure 4d–f). Indeed, less bubble-shaped pores are observed on the edges of the columns, and the CMAS seems to have reacted with a smaller depth of the co-stabilised coatings compared with 8YSZ. The coating also looks brittle with several cracks of columns irrespective of the amount of deposited CMAS since the cracks were also observed in the annealed coatings without CMAS.

**Figure 5:**
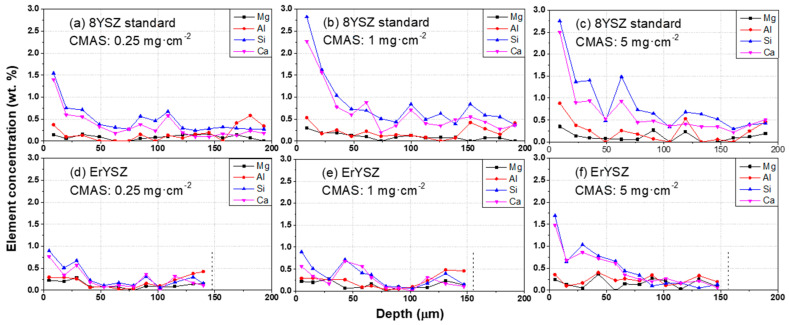
Energy-dispersive spectrometry (EDS) elemental analysis of the CMAS compounds present in the cross-sections of (**a**–**c**) standard 8YSZ and (**d**–**f**) co-stabilised ErYSZ after annealing at 1250 °C for 1h with (**a**,**d**) 0.25 mg·cm^−2^ (**b**,**e**) 1 mg·cm^−2^ and (**c**,**f**) 5 mg·cm^−2^ of CMAS deposits.

The EDS elemental analysis of the cross-sections of the 8YSZ and ErYSZ coatings (Figure 5) shows that the high concentration of the CMAS elements in the upper part of the coatings decreases with increasing depth.

In the case of 8YSZ standard coatings, CMAS fully penetrated the coatings (Figure 5a–c). In contrast, very low contents of the CMAS constituent elements were detected deep in the ErYSZ coatings (Figure 5d–f) even when the maximum amount of 5 mg·cm^−2^ of CMAS had been deposited. This means that the penetration of the CMAS melt was limited in the ErYSZ coating.

**Figure 6:**
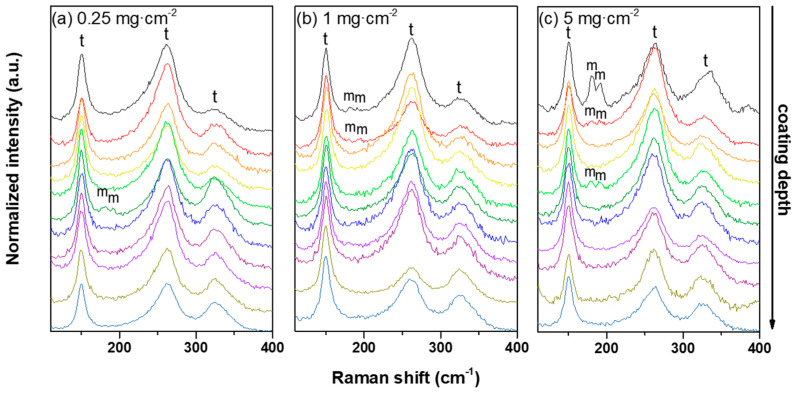
Raman spectra in the cross-sections of the standard 8YSZ coatings with CMAS deposits of (**a**) 0.25 mg·cm^−2^, (**b**) 1 mg·cm^−2^, and (**c**) 5 mg·cm^−2^ annealed in air at 1250 °C for 1 h.

Figure 6 shows the phase transformations of zirconia of the 8YSZ standard coatings exposed to different amounts of CMAS.

**Figure 7:**
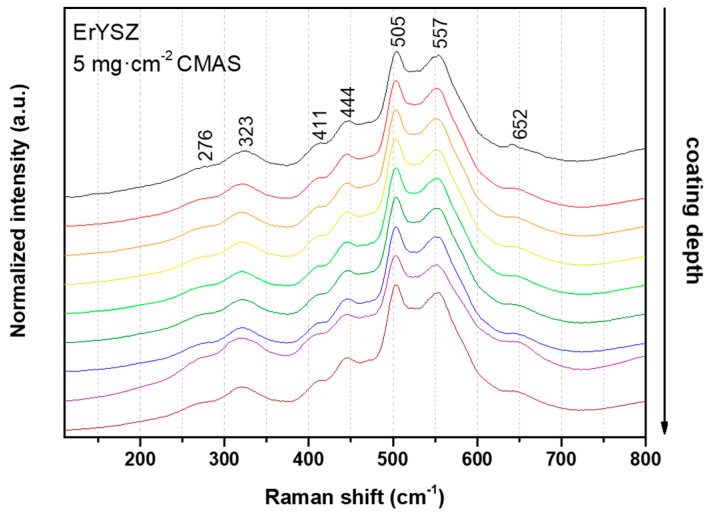
Raman spectra in the cross-sections of the ErYSZ coating after CMAS attack with a 5 mg·cm^−2^ deposit.

The Raman spectra along the cross-section indicate little transformation of the initial tetragonal phase of zirconia (*t*′-ZrO_2_) into the Y-lean monoclinic phase (*m*-ZrO_2_). In addition, the transformation does not seem to depend on the amount of deposited CMAS and only a very minor presence of the *m*-ZrO_2_ occurred randomly when increasing the CMAS deposit. In contrast, no particular phase transformations seem to have occurred in any of the ErYSZ coatings attacked by CMAS, since all the Raman spectra were identical to the as-deposited one (see Figure 7).

**Figure 8:**
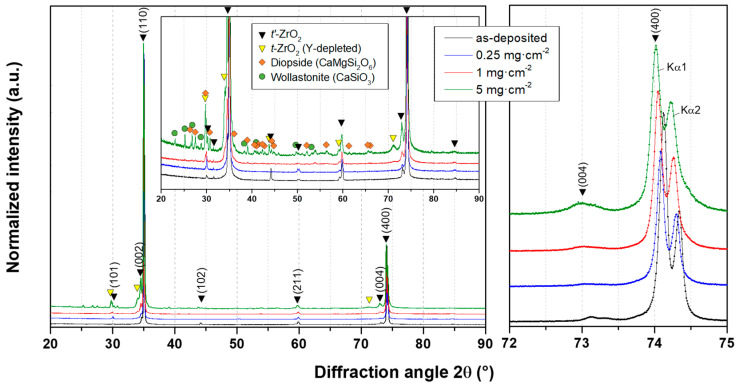
X-ray diffractograms of the 8YSZ standard coatings in their as-deposited conditions and after annealing in air at 1250 °C for 1 h with the different CMAS deposits.

The X-ray diffractograms of standard 8YSZ coatings before and after CMAS attack (Figure 8) show that the main peaks for stabilised tetragonal zirconia (*t*′-ZrO_2_: ICSD 173961) are the (110) and (400) planes.

After the annealing at 1250 °C with the CMAS deposits, the recrystallisation of ZrO_2_ is evidenced by the growth of the (101) and (004) peaks. Moreover, other smaller peaks that can also be attributed to a tetragonal phase of zirconia are appearing for the (101), (110), (211), and (004) planes with a slight shift towards lower angles indicating an increase of the cell unit parameter of this recrystallised *t*-ZrO_2_. When taking a closer look at peaks between 72° and 75°, one can note that the (400) peaks are slightly drifting towards lower angles and tend to broaden as the deposited amount of CMAS increases. However, no peaks of the monoclinic phases of zirconia (*m*-ZrO_2_: ICSD 89426) could be observed on the diffractograms despite the observation of the characteristic doublet with Raman spectrometry (Figure 6). In addition, with the higher deposited amount of CMAS, recrystallised phases of diopside (CaMgSi_2_O_6_: ICSD 17045) and wollastonite (CaSiO_3_: ICSD 33702) are detected on the surface of the 8YSZ coating sample with 5 mg·cm^−2^ of CMAS. 

**Figure 9:**
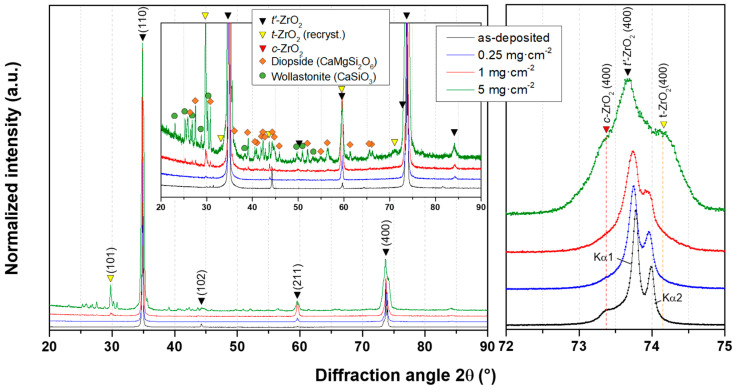
X-ray diffractograms of the co-stabilised ErYSZ coatings in their as-deposited conditions and after annealing in air at 1250 °C for 1 h with different amounts of CMAS deposit.

For the ErYSZ coatings (Figure 9), the results are quite similar as new peaks arise after annealing with CMAS due to the recrystallisation of *t*-ZrO_2_ and as other peaks grow in comparison with the main (110) peak of the as-deposited conditions.

The first difference observed when compared with 8YSZ standard composition lay in the greater intensity of the peaks standing for the recrystallised phases of CMAS (diopside and wollastonite). Moreover, when focusing on the 72° to 75° range, although the shift of the (400) peaks towards lower angles with the increasing CMAS amount is quite similar to that observed for the standard 8YSZ coatings, the broadening of the peaks seems more significant. In addition, while there is still no evidence of the monoclinic phase of zirconia, the cubic phase (*c*-ZrO_2_) can be observed both in the as-deposited conditions and after annealing with CMAS deposits.

### 3.3. Thermal Diffusivity and Conductivity of the Coatings

**Figure 10:**
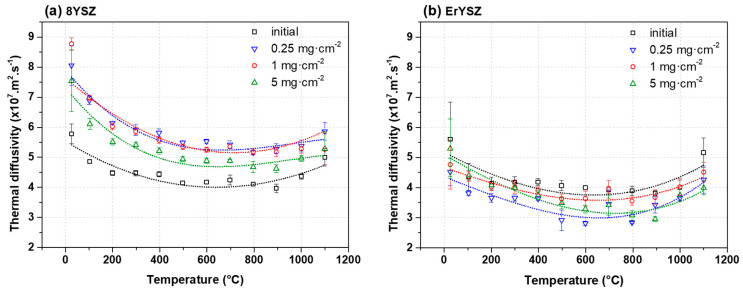
Thermal diffusivity of (**a**) 8YSZ standard and (**b**) ErYSZ thermal barrier coating (TBC) systems in the initial condition and after CMAS attack.

In the initial conditions, the thermal diffusivity of 8YSZ standard coating displays a maximum of ≈5.8 × 10^−7^ m^2^·s^−1^ at room temperature (RT) and a minimum of ≈4 × 10^−7^ m^2^·s^−1^ at high temperature (Figure 10a).

After annealing in air at 1250 °C with CMAS deposits, the thermal diffusivity of the 8YSZ standard coatings increases. With 0.25 and 1 mg·cm^−2^ of CMAS, the thermal diffusivities are equivalent with a maximum of ≈8 × 10^−7^ m^2^·s^−1^ at room temperature and a minimum of ≈5.2 × 10^−7^ m^2^·s^−1^ at high temperature. However, the thermal diffusivity of the coating with 5 mg·cm^−2^ of CMAS is slightly less than the ones with the 0.25 and 1 mg·cm^−2^ deposits. The thermal diffusivity falls to ≈7.5 × 10^−7^ and ≈4.6 × 10^−7^ m^2^·s^−1^ at room and high temperature, respectively.

For the initial ErYSZ coatings (Figure 10b), the thermal diffusivity is lower than 8YSZ standard with a maximum of ≈5.5 × 10^−7^ m^2^·s^−1^ at RT and a minimum of ≈3.8 × 10^−7^ m^2^·s^−1^ at high temperature. However, the thermal diffusivity decreases after the reaction of the coatings with the CMAS deposits. The thermal diffusivity of the coating with CMAS deposit of 0.25 mg·cm^−2^ is the lowest and ranges between ≈2.8 × 10^−7^ and ≈4.5 × 10^−7^ m^2^·s^−1^. The coating exposed to 5 mg·cm^−2^ of CMAS displays intermediate values between ≈5 × 10^−7^ and ≈3 × 10^−7^ m^2^·s^−1^, while the coating exposed to 1 mg·cm^−2^ exhibits values ranging between ≈4.6 × 10^−7^ and ≈3.6 × 10^−7^ m^2^·s^−1^, which are close to those of the initial conditions.

**Figure 11:**
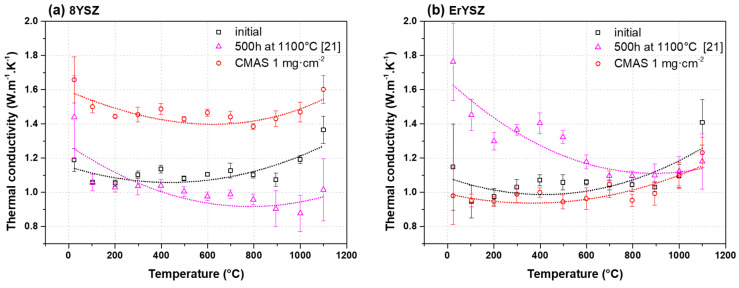
Thermal conductivity of (**a**) 8YSZ standard and (**b**) ErYSZ TBC systems in the initial condition and after CMAS attack with 1 mg·cm^−2^ of CMAS deposit compared with aged coatings at 1100 °C for 500 h from previous work [21].

For comparison purposes, the calculated thermal conductivity of the 8YSZ and ErYSZ coatings in the initial condition and after the CMAS attack with 1 mg·cm^−2^ is plotted in Figure 11 alongside one of the coatings that had been annealed at 1100 °C for 500 h in a previous study [21].

In the initial conditions, the ErYSZ coating (Figure 11b) displays a slightly lower thermal conductivity than its standard 8YSZ counterpart (Figure 11a) with a slight difference of ≈0.1 W·m^−1^·K^−1^ on the whole temperature range. After the CMAS attack, the 8YSZ coating displays a thermal conductivity between 1.4 and 1.6 W·m^−1^·K^−1^, which is much higher than in the initial condition. In contrast, the ErYSZ coatings annealed with the CMAS deposit do not evolve as significantly as the 8YSZ standard composition and exhibit a similar thermal conductivity as in the initial state. However, the response of thermal conductivity of the two compositions is completely different with the coatings annealed for 500 h (without CMAS) samples. For the 8YSZ coatings, the thermal ageing leads to a thermal conductivity close and even lower at high temperature than in the initial conditions, while for the ErYSZ, the thermal conductivity of the aged coating is significantly higher than in the initial conditions until 600 °C and then, it tends to be equivalent to the one of the initial conditions at high temperature.

## 4. Discussion

Figure 1 demonstrates that annealing of both the 8YSZ standard and the ErYSZ coatings at 1250 °C in air for 1 h results in microstructural transformations including sintering of the small intra-columnar pores and the closing of the pores trapped in the feather-like structure together with the growth of a thick *α*-Al_2_O_3_ TGO (see Figure 1). Similar transformations were observed in previous works for standard EB-PVD YSZ coatings [29,30,31] and ErYSZ coatings [21]. However, no phase transformation is highlighted after the annealing treatment, as only the tetragonal phase of zirconia is detected in the Raman analysis (see Figure 2). 

After the exposure of the coatings to the CMAS deposits for 1 h at 1250 °C, some residues of the glassy melt are found at the surface of the coatings (see Figure 3). This suggests that either the whole amount of CMAS deposited did not melt completely or that it was too viscous to fully penetrate in the coatings. The first hypothesis can probably be disregarded because of the glassy appearance of the residues. While studying the effect of the columnar coating morphology on CMAS infiltration resistance, Naraparaju et al. showed that a feathery structure of the columns (similar to the one observed for our coatings, see Figure 1) could hamper the infiltration of the glassy melt due to a high tortuosity associated to smaller intercolumnar gaps [32]. Nevertheless, the capillaries formed by the columnar structure of the EB-PVD coatings still allowed the CMAS to penetrate the standard YSZ coatings (see Figure 5a–c) and to sinter the columns (see Figure 3d–f). 

**Figure 12:**
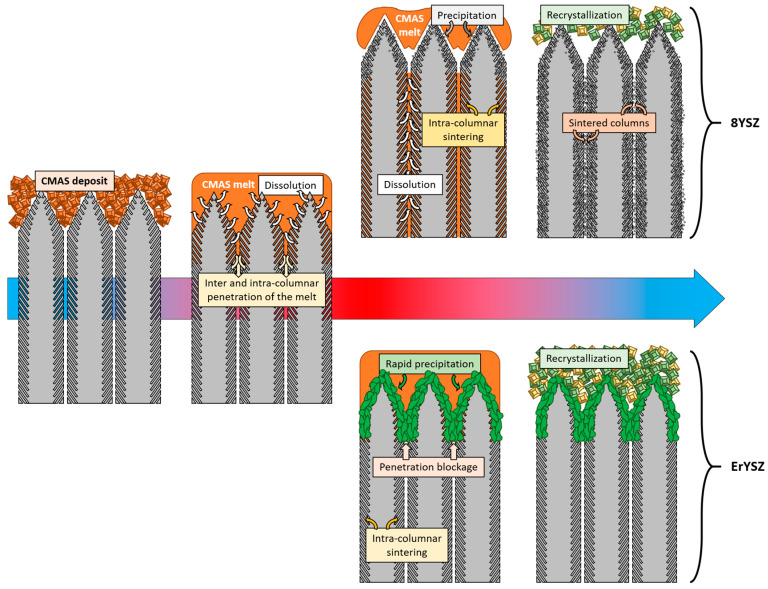
Schematic drawing of the CMAS interaction with the 8YSZ and ErYSZ columnar coatings during the annealing at 1250 °C (1 h) and final cooling.

The evolution of 8YSZ and ErYSZ EBPVD coatings exposed to CMAS is summarised in Figure 12.

For both coatings, the coating material dissolved in the CMAS as it started to melt and wet the surface of the columnar coating. For the 8YSZ standard composition, the dissolution of the metastable *t*′-ZrO_2_ led to a Zr saturation of the CMAS melt as previously described by Krämer et al. [33]. Then, different structures of zirconia can precipitate from this melt due to the difference of solubility of Y^4+^ and Zr^3+^ cations in the CMAS, i.e., either fully stabilised zirconia with an Y-enriched composition compared with the 8YSZ initial composition or else, Y-depleted zirconia. Krämer et al. also showed with a simple model that the CMAS melt could penetrate a columnar coating of 200 μm in less than one minute at 1240 °C. Thus, the CMAS melt could interact with the 8YSZ columns along the full depth of the coating as also observed in our case (Figure 5**)**. Therefore, upon cooling, the residual CMAS and the precipitated new phases of zirconia start to recrystallise into new phases. As observed in this work by XRD in Figure 8, the residual CMAS recrystallise into diopside and wollastonite, while a Y-depleted phase of zirconia is also observed with the peaks of *t*-ZrO_2_ at a lower diffraction angle compared to the one of *t*′-ZrO_2_ from the 8YSZ initial composition. Since the melt fully penetrated the coating, the recrystallised phases of CMAS and zirconia tend to fill the initial voids between the columns as well as the feathery microporous structure of the column itself (inter and intra-columnar sintering, respectively). This mechanism of dissolution/reprecipitation was also observed in several other studies on the interaction of the 8YSZ material with various compositions of CMAS and volcanic ashes [12,34,35,36,37,38]. However, for the ErYSZ coating, the precipitation kinetics of new phases is fostered by the presence of Er. As stated previously, rare-earth zirconates coatings were shown to foster the rapid crystallisation of stable apatite and fluorite phases as soon as the CMAS melt starts to dissolve the ceramic coating [7,20]. Although no fluorite nor apatite phases were observed using XRD, a significant phase transformation of zirconia was shown in Figure 9 with the appearance of Y-depleted *t*-ZrO_2_ as well as fully stabilized *c*-ZrO_2_. Krämer et al. also showed in another study that thanks to the presence of rare earth, the penetration of CMAS did not exceed a depth of 30 μm [13]. Therefore, for the ErYSZ coatings of this study, the presence of Er could foster the rapid formation of precipitates that thus hinder the penetration of the CMAS melt, as shown in Figure 5, where CMAS components are hardly detected below 75 μm, i.e., half the thickness of the TBC.

The sintering phenomenon, mostly due to CMAS infiltration in our case, is a well-known factor that reduces the thermal insulation of EB-PVD coatings [30,31] and can therefore explain the increase of the thermal diffusivity of the 8YSZ coatings exposed to CMAS. In a previous work, similar CMAS deposits (0.25 to 3 mg·cm^−2^) were used on freestanding plasma-sprayed YSZ coatings, and an increasing trend of the thermal diffusivity was observed with increasing CMAS deposits [22]. However, such a clear increasing tendency is not observed for the EB-PVD coatings in this work. In contrast, even lower thermal diffusivities are observed for the higher amounts of CMAS, i.e., the lowest thermal diffusivity occurs with the greatest amount of deposited CMAS (5 mg·cm^−2^) (Figure 10a). Thus, the thermal diffusivity in this case does not seem to be directly related to the amount of deposited CMAS.

This suggests that additional factors other than sintering could be responsible for the change in the heat transport properties of the TBCs exposed to CMAS. These include (i) the phase transformations of zirconia [31,39,40], (ii) the thermal transport properties of the residual glassy melt of the CMAS or its reaction products [41], and (iii) the increase of interfacial resistance [21,42].

The phase transformations of YSZ in contact with CMAS have been shown to result from the depletion of Y in the YSZ via the dissolution–reprecipitation mechanism of corrosion by molten CMAS [7,11,33]. Bisson et al. showed that decreasing the Y_2_O_3_ content in YSZ single crystals contributed to an increase in the thermal diffusivity [39]. However, only minor peaks resulting from the recrystallisation of *t*-ZrO_2_ are observed in the diffractograms (Figure 8). Thus, the increase in thermal diffusivity due to these phase transformations is likely to be negligible, as already stated in our previous study [22].

Considering the thermal transport properties of CMAS itself, Kakuda et al. showed that the thermal diffusivity of crystallised CMAS was very close to that of the fully dense 7YSZ and that the diffusivity of amorphous CMAS was half that of its crystallised form [41]. Moreover, Karamanov and Pelino showed that the formation of diopside and wollastonite glass ceramics (detected via XRD Figure 8 and Figure 9) caused the development of significant crystallisation-induced porosity (≈10 vol.%) due to the crystallisation volume variation of diopside and wollastonite [43]. Therefore, the addition of CMAS in the coating should lead to a decrease of the thermal diffusivity, and the presence of residual CMAS on top of the coatings would also hamper the heat transport. Thus, this would explain the smaller thermal diffusivity values of the 8YSZ coating exposed to 5 mg·cm^−2^ of CMAS deposit, which was almost entirely covered by CMAS.

The growth of the TGO observed in the case of the annealed coatings (see Figure 1) might also contribute to resistance to thermal transport, leading to an underestimation of the coating’s thermal diffusivity because the TGO growth has not been considered in the multilayer calculation. The influence of the TGO was studied in previous works when measuring the thermal diffusivity of plasma-sprayed coatings as part of a complete TBC system (substrate + bond coating + top coating) [42]. In addition, after 500 h annealing at 1150 °C of the same standard YSZ EB-PVD coating, the increase in TGO thickness from 0.6 to 1.2 µm already led to a discrepancy between the thermal diffusivity of coatings of different thicknesses; i.e., a 5% decrease of the top coating thickness was linked to a 10% decrease of the calculated thermal diffusivity values [21]. Thus, the underestimation of the thermal diffusivity would be greater for the thinner coatings. 

As a matter of fact, the 8YSZ coating exposed to 1 mg·cm^−2^ CMAS deposit is 3% thinner than the one exposed to 0.25 mg·cm^−2^ CMAS deposit (Table 1), which displays a slightly lower thermal diffusivity than the latter (Figure 10a) despite the greater sintering observed in Figure 3. Moreover, the standard coating annealed with 5 mg·cm^−2^ of CMAS, which is 15% thinner than the one with 0.25 mg·cm^−2^ of CMAS, shows a significantly lower thermal diffusivity (Figure 10a). Therefore, the lower calculated thermal diffusivity values could be assigned to both the smaller thickness of the top coating that exacerbates the TGO influence and the remaining CMAS on top of the coatings.

In the case of the ErYSZ coatings, the thermal diffusivity in the initial conditions is observed to be smaller than that of standard 8YSZ coating (Figure 10b). This agrees with the effect of erbia doping that was demonstrated to be responsible for the thermal diffusivity reduction [6,21]. As a matter of fact, the doping of the YSZ coating with Er^3+^ cation introduces more oxygen vacancies as well as strain centres into the lattice (8 mol.% in ErYSZ instead of 4 mol.% in 8YSZ) that reduces the phonon mean free path and hence the heat transport by phonons. In addition, doping with erbia gives a pink colour to the coating, which can also contribute to a reduction of heat transport by reducing the radiation transport in the visible range and thus in the near infrared [6]. When considering the CMAS-attacked coatings, the penetration of the CMAS is in fact limited to the upper part of the coatings (see Figure 4, Figure 5d–f and Figure 12) and only a limited transformation of the microstructure of the top of the columns is indeed observed irrespective of the amount of deposited CMAS. This behaviour was ascribed to the high reactivity of the RE oxide that could mitigate CMAS penetration via a rapid precipitation of fluorite and apatite phases [7]. One shall note that with the addition of such RE oxide, the reaction of the coating material with the CMAS melt could lead to the formation of different phases such as garnet silicate, orthosilicate Ca_3_RE_2_Si_3_O_12_, or disilicate RE_2_Si_2_O_7_, but none is observed in this study [20]. Therefore, as the CMAS penetration was hampered, the impact of the sintering by the melt is limited in the ErYSZ coatings. Thus, in comparison with the 8YSZ standard TBC, the calculated thermal diffusivity of this ErYSZ coating should mostly be affected by (i) the coverage of the surface by CMAS, (ii) the TGO growth whose impact depends on the TBC thickness, and (iii) the phase transformations. The calculated thermal diffusivity of the CMAS attacked ErYSZ coatings was not significantly different from that of the initial condition (Figure 10b). This means that the possible contribution to the increase of the thermal diffusivity by sintering and phase transformations was compensated by the combination of the insulating potential of the residual CMAS and the TGO growth. This hypothesis is supported by the results of Figure 4, Figure 7 and Figure 9, where neither the sintering nor the phase transformations appear significant for all the ErYSZ coatings. 

Therefore, the slight decrease of the thermal diffusivity observed for the CMAS attacked ErYSZ coatings could be ascribed to the insulating potential of both the TGO growth and the residual CMAS as well as the potential reaction products. As the coverage by the CMAS melt was observed to be similar for both ErYSZ and YSZ coatings (see Figure 3 and Figure 4), the insulating potential should be similar. However, its impact might be even slightly higher in the case of the ErYSZ as the CMAS penetration is negligible. In addition, the impact of the TGO growth on the thermal diffusivity is assumed to be similar, since the TGO grown on both the YSZ and ErYSZ coatings are equivalent (from ≈0.6 µm in the initial conditions to ≈2 µm; see Figure 1). Nevertheless, the thickness of the top ErYSZ coating is significantly thinner than the one of the standard YSZ; e.g., there is a ≈36 µm gap between the thinner standard top coating (8YSZ with 5 mg·cm^−2^ of CMAS) and the thicker ErYSZ one (ErYSZ with 5 mg·cm^−2^ of CMAS) (see Table 1). Therefore, since the TGO/coating thickness ratio is lower for the ErYSZ than for the YSZ, the overall impact of the TGO on the thermal diffusivity is greater in the former.

The calculated values of thermal conductivity for the standard 8YSZ coating in its initial state (Figure 11a), which lay between 1.0 and 1.2 W·m^−1^·K^−1^, are in the low range of the thermal conductivity of YSZ EBPVD coatings [44]. As reported by Renteria et al., the effect of the morphology is significant on the resulting thermal conductivity of EBPVD TBCs [29]. Therefore, these particularly low values could be due to the fine and stable porous microstructure of the feathery structure observed on Figure 1. As a matter of fact, a thermal conductivity of ≈1.0–1.1 W·m^−1^·K^−1^ was measured in previous studies on the temperature range between room temperature and 1000 °C for such feathery structured TBCs [44]. The reduction of the thermal conductivity due to erbia doping observed in this work is slightly below the values given by Nicholls et al.; i.e., in this work, the ≈0.1 W·m^−1^·K^−1^ difference between YSZ and ErYSZ correspond to a reduction of ≈9% while Nicholls et al. observed a reduction of ≈20% [6]. This difference can be explained by the errors in the calculated values of thermal diffusivity that arise from the difference in coating thickness between YSZ and ErYSZ coatings (≈50 µm). After 500 h of annealing at 1100 °C, the thermal conductivity of the 8YSZ coating does not increase as expected, but it is calculated to be even lower than the coating in its initial state. This phenomenon was explained in a previous study to be due to the increase of interfacial resistance as the TGO grows during the annealing and the fact that its contribution is disregarded with the 2-layer calculation model that hence leads to the underestimation of the thermal diffusivity true values [21]. Thus, the thermal conductivity of the annealed 8YSZ coating is lower than the one reported by Rätzer-Scheibe et al., where the thermal conductivity of YSZ coatings was observed to increase of ≈0.5 W·m^−1^·K^−1^ after 500 h of exposure to 1100 °C in air [30]. However, the annealing of the ErYSZ coatings led to a significant increase of the thermal conductivity that was proposed to result from the greater sensitivity of the Er-doped coatings to sinter, hence resulting in a more significant loss of the thermal insulating potential [21].

Furthermore, the thermal conductivity of the two types of coatings evolves differently after the annealing with the CMAS deposits in comparison with the annealing without CMAS. For the 8YSZ coating, the calculated thermal conductivity reaches values between ≈1.6 and 1.4 W·m^−1^·K^−1^ between RT and 1100 °C, which is about one-third higher than its initial thermal conductivity. Considering the potential underestimation of the interfacial resistance in the thermal diffusivity calculations, the increase of the true thermal conductivity could be even more important. Nonetheless, the thermal conductivity is still lower than that of some other YSZ coatings, with or even without ageing treatment, measured in different studies [30,44,45]. However, for the ErYSZ coatings, the CMAS attack did not significantly change the thermal conductivity results compared to the coating in the initial state.

## 5. Conclusions

This study investigated the degradation potential of minor CMAS deposits (0.25, 1.0, and 5.0 mg·cm^−2^) on ErYSZ and standard 8YSZ EB-PVD TBCs annealed at 1250 °C for 1 h. CMAS did not fully penetrate the coatings, which resulted in the partial coverage of the surface of the coatings. Irrespective of the CMAS amount, the melt that partly penetrated the ceramic layer reached the full thickness of the standard YSZ coatings but was limited for the ErYSZ coatings. Thus, the associated microstructural and chemical transformations were less important for the ErYSZ coatings, hence resulting in a better thermal insulation after ageing in the presence of CMAS.

## Figures and Tables

**Table 1:** Samples of the study.

Sample ID	Initial Coating	TBC Thickness (µm)	CMAS Deposit (mg·cm^−2^)
As-deposited	8YSZ standard (ZrO_2_—8 mol.% Y_2_O_3_)	202.0 ± 5.6	-
Std025		209.1 ± 1.5	0.26 ± 0.1
Std1		203.0 ± 1.1	1.03 ± 0.1
Std5		194.4 ± 1.7	5.07 ± 0.1
As-deposited	ErYSZ (ZrO_2_—4 mol.% Y_2_O_3_—4 mol.% Er_2_O_3_)	148.3 ± 8.3	-
Er025		145.7 ± 1.9	0.25 ± 0.1
Er1		155.9 ± 2.4	0.98 ± 0.1
Er5		158.2 ± 1.2	5.02 ± 0.1
